# Investigation of Relationship Between Social Capital and Quality of Life in Multiple Sclerosis Patients

**DOI:** 10.5539/gjhs.v6n6p261

**Published:** 2014-08-15

**Authors:** Shahnaz Rimaz, Kazem Mohammad, Maryam Dastoorpoor, Ensiyeh Jamshidi, Reza Majdzadeh

**Affiliations:** 1 Department of Epidemiology, School of Public Health, Iran University of Medical Sciences, Tehran, Iran; 2 Department of Epidemiology and Biostatistics, School of Public Health, Tehran University of Medical Sciences, Tehran, Iran; 3 Research Center for Modeling in Health, Institute for Futures Studies in Health, Kerman University of Medical Sciences, Kerman, Iran; 4 Community Based Participatory Research Center, Iranian Institute for Reduction of High-Risk Behaviors, Department of Health Education and Promotion, School of Public Health, Tehran University of Medical Sciences, Tehran, Iran; 5 Department of Epidemiology and Biostatistics, School of Public Health, Knowledge Utilization Research Center, Tehran University of Medical Sciences, Tehran, Iran

**Keywords:** social capital, quality of life, Multiple Sclerosis, MSQOL-54

## Abstract

**Background:**

A large portion of existing medical research on Multiple Sclerosis patients focuses more on predicting medical variables (such as diagnosis, treatment) and individual variables such as the onset of disease, gender, etc., rather than broader socio-contextual factors. So that, here has yet been no study investigating factors such as social capital in Multiple Sclerosis patients.

**Aim:**

The purpose of this study is determining the relation between social capital and quality of life in Multiple Sclerosis patients who referred to Iran Multiple Sclerosis Society in 2012.

**Method:**

This cross-sectional study was conducted on 172 patients visiting Iran Multiple Sclerosis Society (Tehran) during 10 months via convenience samplings and face to face interviews. Tools for collecting data included World Bank’s social capital integrated questionnaire (SC-IQ) and Multiple Sclerosis Quality of Life (MSQOL) -54.

**Results:**

The average age of patients was 34/8±9/6. The analysis of the six dimensions of social capital questionnaire showed that the highest average score belonged to membership in groups and networks (63/3±15/3) and the lowest one was about trust and solidarity (44/3±13/7).The results of the regression model showed that there is a statistical significant and positive relation between social capital and quality of life (P > 0.0001).

**Conclusion:**

Since the present study has been conducted for the first time in this vulnerable subpopulation of patients, its results can provide invaluable information regarding the quality of life and at the same time present hypotheses about the contributing factors.

## 1. Background

Multiple Sclerosis (MS) is a chronic, progressive, and a common demyelination disease of the central nervous system which results in decreasing individual and social performance (Murray, 2009). Currently there are more than 1.3 million individuals all over the world suffering from this disease (Kingwell et al., 2012). Studies conducted in the Middle East and Iran in recent years indicate the high and increasing spread of MS in these areas where the incidence rate of this disease increased significantly from 0.68 in 1989 in 2.93 in 2008 among 100 thousand individuals (Elhami, Mohammad, Sahraian, & Eftekhar, 2011).

MS patients encounter problems related to their disease that restrict their participation in health-related activities. As a result, subsequent consequences of their disease increase and they face limitations in their independent life which eventually exert negative influence on the quality of their lives (Morgante, 2000). There is no doubt that one of the appropriate solutions for enhancing man’s quality of life is identifying the contributing factors which influence people’s quality of life. A large portion of existing medical research on the quality of life in Multiple Sclerosis patients focuses more on predicting medical variables (such as diagnosis, disease progression type, and treatment) (Polman et al., 2011; Weinshenker et al., 1991), individual variables (such as the onset of disease, gender, family record, social and individual supports, co morbid diseases, health-related behaviors, and other confounding factors (Marrie et al., 2009; Masterman et al., 2000; Schwartz & Frohner, 2005) rather than broader socio-contextual factors.

It seems that there has yet been no study investigating the relation between socio-contextual factors such as social capital and quality of life in Multiple Sclerosis patients. Features of social environment in which patients live and the degree of their social interactions such perception of trust and social cohesion in their own neighborhood or the availability local community based organizations and social participation can be effective on the outcomes of patients’ quality of life (Petersen, 2008). In other words, the quantity and quality of social relations and interactions which have found a new aspects using the social capital concept and received great attention have a profound effect on the quality of life of individuals in terms of objective and mental quality of life (Davis, 1992).

Despite the diversity in definitions, social capital is extensively defined as the degree of social cohesion, interactions, trust, mutual reciprocity, perception and feeling of mutual commitment among members of a social group (R. Putnam, 2012; R. D. Putnam, 1995). Social capital has two major components: cognitive component and structural component. The cognitive component includes norms, values, attitudes, and shared beliefs and it’s defined as the individuals’ perception of the level of trust, mutual reciprocity, social cohesion, social commitments and interactions among social groups (R. Putnam, 2012; R. D. Putnam, 1995). The structural component includes social supports which facilitate human interactions and frequently refers to the intensity and density of the ties of sociability, social participations, social institutions, or patterns of civil interactions (Araya et al., 2006; Harpham, Grant, & Thomas, 2002).

Social capital is one of the factors which, in health studies, is known as a contributing individual and social factor effective on health (Islam, Merlo, Kawachi, Lindström, & Gerdtham, 2006). Different national and international studies have revealed that social capital components especially the dimension of social trust have a remarkable effect on people’s health which cannot be solely justified by economic criteria (Helliwell, 2006). Although studies have confirmed that there is a positive relation between social capital (measuring via different tools) and a health related variables in widespread populations (such as the causes of mortality (Wen, Cagney, & Christakis, 2005), self-rated health, incidence of diseases like coronary artery diseases (Scheffler et al., 2008), sexual transmitted diseases (Holtgrave & Crosby, 2003), health-related behaviors including smoking (Brown, Scheffler, Seo, & Reed, 2006), physical activities, eating habits and obesity (Poortinga, 2006), birth weight (O’Campo, 2003), child development (Drukker, Kaplan, Feron, & Van Os, 2003) and mental health (Araya et al., 2006) it seems social capital advantages have not been sufficiently studied in health tradition on the outcomes of the quality of life of the public or among vulnerable subpopulations such as MS patients.

Thus, investigating social capital and its effect on the quality of life among MS patients deems necessary from two aspects. On the one hand, MS patients may be affected by the neighborhood structure since they spend more time at home and rely more on local socio-structural resources and on the other hand, disabilities and co morbid conditions in MS patients can reduce their membership and participation in groups and communities. Therefore, enhancing their interactions and social participation will both help the society use their abilities and improves their mental and physical health capacities and quality of life. As a result, since social capital is an important strategy to increase social participation of MS patients in the neighborhood and enhance their social mobility and consequently their quality of life, this research aims to determine the relation between social capital and the quality of life in MS patients and take a critical step in designing proper interventions to enhance social capital and as a result the quality of life.

## 2. Method

### 2.1 Study Population and Sampling

Following the approval of the ethics committee of Tehran University of Medical Sciences and doing the required arrangements, this cross-sectional study was carried out on 172 patients who referred to Iran Multiple Sclerosis Society in Tehran during 10 months from April 2012 to February 2013. Since the population referring to the Iran Multiple Sclerosis Society was limited, samples in this study were selected among volunteer patients through simple and non-probability sampling. Having a letter of introduction, the interviewer visited the research setting for sampling every day except Fridays (weekend) and once permitted orally and in a written consent form by those MS patients volunteer to take part in the study, she kept on sampling until he got to the required sample size.

The inclusion criteria were as the following: Multiple Sclerosis patients based on Poser criteria (Poser & Brinar, 2001), patients ranging from 15-65 years old, willing to take part in the study, from all MS types: primary progressive MS, secondary progressive MS, and relapsing MS, recovery and disability score (EDSS) mild (0-3) to moderate (3.5-6.5). The reason was that researchers wanted to make sure that patients could take part in the study and were able to visit the MS Society.

### 2.2 Research Tools Included Following Questionnaires

1) *Demographic Information Questionnaire* including age, gender, marital status (married, single, widowed, divorcee), literacy (illiterate, elementary, high school, university), number of successful completed academic years, employment (employed, unemployed), average income of the individual and family, area of the house (approximate index of economic status), number of current members of the family (including the participant), number of children, ownership of the house (landlord, tenant), number of owned rooms (objective index of social welfare), duration of stay in the house, ethnicity, being health insured, duration of suffering from MS, the onset of the disease, the onset of the first symptoms of the disease, first signs and symptoms (sensory, kinesthetic, visual), family record of MS, other diseases (yes, no), current use of medications (Corticosteroids), rehabilitation treatments, using complementary and alternative remedies (yoga, sports, meditation, dietary and using herbs, energy medicine and relaxation, acupuncture and acupressure and their efficacy (absolutely ineffective, fairly effective, highly effective), disease progression type (relapsing-remitting, primary and secondary progressive).

2) *Multiple Sclerosis Quality if Life-54 Instrument* based on 36-Item Short Form Health Survey (SF-36) that its validity and reliability were confirmed in Iran so that the reported Cronbach’s alpha coefficient by Ghaem et al. for this tool was 0.96 (Ghaem, Borhani Haghighi, Jafari, & Nikseresht, 2007). This questionnaire included 54 questions among which 18 questions were in 14 dimensions specific to MS patients (Physical Health, role limitations due to physical problems, role limitations due to emotional problems, social function, health distress, sexual function, satisfaction with sexual function, pain, energy, health perception, overall quality of life, change in health, cognitive function, and emotional well-being) and 36 questions referred to general quality of life. The questions had 2 to 7 choices on Likert scale. Finally, the score of patient’s quality of life was calculated by the scores of the two combined dimensions. These two combined dimensions were physical health and Mental Health. The scores of all 14 dimensions as well as the two combined dimensions ranged from 0 to 100. Higher scores indicate a better condition (Vickrey, Hays, Harooni, Myers, & Ellison, 1995).

3) *World Bank’s Social Capital Integrated Questionnaire*. This questionnaire has been designed for developing countries and studies social capital at the level of families (Grootaert, Narayan, Jones, & Woolcock, 2004). Evidence shows this tool can provide useful and abundant information (Iisakka, 2006). The Iranian version of this questionnaire was designed by Nedjat et al. in 2013 and included 39 main questions and six dimensions: (1) Groups and networks, (2) Trust and solidarity, (3) Collective action and cooperation, (4) Information and communication, (5) Social cohesion and inclusion, (6) Empowerment and political action. In this research, every dimension included 7, 7, 3, 2, 12, 8 questions respectively on Likert scale, multiple-choices and also yes-no questions (Nedjat, Majdzadeh, Kheiltash, Jamshidi, & Yazdani, 2013). It is noteworthy to mention that the score for each dimension ranges from 0 to 100 and higher scores indicate higher social capital. The approximate time to fill out a questionnaire for every individual was about 25-30 minutes.

Questionnaires were filled out by the researcher before and after classes and during breaks in the hall used for educational classes and also in the physiotherapy section in the form of face to face interviews and without any additions or deletions on the part of the researcher.

### 2.3 Data Analysis

In this research, to test the hypotheses and also provide answers to research questions, first of the normality of the data was investigated using Kolmogorov-Smirnov test. The results of the test confirmed the normality of data. Statistical indexes such as mean and standard deviation were used for the average score of social capital, quality of life and its dimensions. To investigate the correlation between independent variables and quality of life for each dimension, Pearson correlation coefficient, one-way ANOVA, and independent two-sample t tests were used (variables whose p-value was below 0.2 entered regression model). Finally, backward multiple linear regression was used to detect the effect of independent variables such as social capital on combined dimensions of quality of life (physical health and psychological health) and to adjusting the effect of confounding factors. It is worthy to note that variables concerning age, monthly income of the patient, monthly income of the patient’s family, number of owned rooms, area of house entered quantitatively and level of education, employment, insurance coverage, record of diseased except MS, ownership of the house, first symptoms of the disease, and alternative treatments entered nominally. Data were analyzed using SPSS statistical software version 20 and the level of significance was 0.05.

### 2.4 Ethical Considerations

Ethical issues (including plagiarism, informed consent, research misconduct, data fabrication and/or falsification, double publication and/or submission, redundancy, etc.) have been completely observed by the authors. Informed consent (oral and written) of all participants was obtained and a regulation of the Declaration of Helsinki was followed throughout the study.

## 3. Results

### 3.1 Demographic-Socioeconomic and Clinical Factors of Participants

The participants of this research ranged between 34/8±9/6 years old, the average age for the outbreak of the first symptoms was 24/9±8/7years old and the duration of the disease was 8/1±6/4. Most participants were women (71.5 percent), with university degree (46.5 percent), single (45.9 percent) and unemployed (69.8). The average area of the house was 102.2±52.9 square meters (Table 1).

**Table 1:** Demographic status of participants

Age	34.8	9.6	16	66

House area (m2)	102.6	52.9	40	450

Duration of stay in neighborhood (Year)	9.9	9.8	1	60

**Variable**	**Frequency**		**Percent**	

**Sex**				

Male	49		28.5	
Female	123		71.5	

**Education status**				

Illiterate	2		1.2	
Elementary school	4		2.3	
Guidance school	9		5.2	
High school	77		8.44	
University	80		46.5	

**Marital status**				

Single	79		45.9	
Married	72		41.9	
Widow/widower	4		3.2	
Divorced	17		9.9	

**Employment status**				

Employed	52		2.30	
Unemployed	120		8.69	

**Ownership status**				

House Owner	108		62.8	
Tenant	64		2.37	

#### 3.1.1 Quality of Life Dimensions

The results of analyzing 14 dimensions of quality of life showed that the highest average score for patients’ quality of life belonged to social function dimension (71.7±21/02) and the lowest one belonged to the role limitations due to physical problems (44/2±39/4). Also, in the combined dimensions of the quality of life the average of mental health (59/8±21/7) was a bit more than that of the physical health (58/2±17/5). And there was a strong correlation between these two dimensions (P < 0.0001) (Table 2).

**Table 2:** Means and standard deviations for quality of life and social capital variables among participants

Quality of life Dimensions	Mean	Standard Deviation	Lower limit	Upper limit
Physical Health	59.9	26.4	0	100
Role limitations due to physical problems	44.2	39.4	0	100
Role limitations due to emotional problems	51.5	42.6	0	100
Pain	70.7	24.5	0	100
Emotional well-being	58.5	23.1	0	100
Energy	58.5	22	0	100
Health perception	61.6	23.4	0	100
Social function	71.7	21.0	7	100
Cognitive function	64.3	25.5	0	100
Health distress	69.9	26.4	0	100
Sexual function	66.2	33.2	0	100
Change in health	4.60	28	0	100
Satisfaction with sexual function	2.53	33	0	100
Overall quality of life	5.65	4.22	0	100
Physical Health Composite Score	5.17	2.58	5.15	92
Mental Health Composite Score	7.21	8.59	7.9	100

**Social Capital Dimensions**				

Groups and networks	63.3	15.3	21.4	96.4
Trust and solidarity	44.3	13.7	6.8	75
Collective action and cooperation	53.2	20	0	100
Information and communication	52.6	20	5.9	94.1
Social cohesion and inclusion	54.1	10.3	25.7	80
Empowerment and political action	47.1	16.7	1.1	94.3

#### 3.1.2 Social Capital Dimensions

In social capital dimensions the highest and lowest average scores belonged to groups and networks (63/3±15/3) and trust and solidarity (44/3±13/7) respectively (Table 1). As it was observed, the average scores in membership in groups and networks, social cohesion and collective action and cooperation dimensions were above 50. In trust and solidarity and empowerment and political action dimensions, however, it was rather serious and patients’ average scores in these dimensions were not above 50.

#### 3.1.3 Quality of Life in Association With Demographic-Socioeconomic-Clinical and Social Capital Variables

To determine the effective factors on the two combined dimensions of physical health and mental health (after reducing the confounding effect of other variables) backward multiple linear regression model was used. Table 3 shows the variables which have a significant relation with the change of outcome or Likelihood-ratio test has not allowed their deletion. As it is shown, the existence or lack of existence of a comorbid disease and consuming Corticosteroids (including Betaferon, Avonex, Rebif) are effective on the score of combined dimension of physical health and dimensions of membership in groups and networks, collective action and cooperation, and empowerment and political action. The existence or lack of existence of a comorbid disease and complementary and alternative treatments (such as yoga, sports, meditation, dietary and using herbs, energy medicine and relaxation, acupuncture and acupressure) are effective on the combined dimension of mental health. Eventually, the research found a statistically significant and positive relation in investigating the relation between the overall quality of life (dependent variable) and overall social capital (independent variable) (P-value>0.0001, β = 2/56). In other words, it seems that every development and improvement in overall social capital leads to increasing overall quality of life.

**Table 3:** Effect of demographic and clinical variables and social capital dimensions on combined constructs of physical and physiological health of quality of life using backward multiple linear regression model

	Residual variables in final model	Standardized B	p-value
	
**Combined dimension of physical health**	*Constant*		**0.08**
	Education Status	**0.24**	**0.06**
	Duration of disease	**-0.25**	**0.05**
	Comorbid disease	**0.32**	0.02*
	Ownership of a house	**-0.27**	**0.06**
	Corticosteroids consumption	**0.37**	0.01*
	Social cohesion and inclusion	**0.25**	**0.09**
	Empowerment and political action	**0.23**	**0.09**

	*Variable*	**Standardized B**	**p-value**
	
**Combined dimension of psychological health**	*Constant*		**0.82**
	Groups and networks	**0.38**	0.02*
	Collective action and cooperation	**-0.53**	0.01*
	Empowerment and political action	**0.50**	0.01*
	Comorbid disease	**0.45**	0.00*
	Unusual treatment other than conventional medicine	**-0.33**	0.03*

## 4. Discussion

As Lin categorizes the results of social capital with the titles of useful results (asset, power, fame) and meaningful results (physical health, psychological health, and satisfaction with life), different studies indicate that improving and advancing the level of social capital can end in the expected quality of life and wellbeing (Lin, 2001).

Our aim of this research was to determine the quality of life and its relation with social capital in a group of MS patients. The results of the present research, in line with similar studies (Drukker et al., 2003; Kim & Kawachi, 2007; Nilsson, Rana, & Kabir, 2006; Petersen, 2008) showed that there is a positive and direct relation between the dimensions of the quality of life and social capital. In other words, it seems that the increase in social capital leads to the enhancement and improvement of the quality of life in Multiple Sclerosis patients and vice versa. The close relation between these two indexes indicates that the improvement of any dimension leads to the improvement of the other one. Given the documents and studies in the field of social capital, one can contend that providing and enhancing social capital is a possible issue. In fact, by understanding the concept of social capital and its importance and consequently acquiring the necessary skill to create it in different contexts of the society, one can observe its strong effect on the dimensions of the quality of life of the public especially vulnerable subpopulations of the society such as MS patients.

In this research, the results of analyzing the combined dimensions of the quality of life showed that the average score of mental health is only a bit more than physical health which is in line with the study of Ghaem et al. (Ghaem & Haghighi, 2008) in Iran which reported the same small difference. This indicates the importance of the psychological components of the quality of life in MS patients. It doesn’t seem that psychological symptoms and disorders such as depression and stress are closely related to physical dimension such as the intensity of disability in MS (Bal, Vázquez-Barquero, Pena, Miro, & Berciano, 1991). Studies have shown that the score of physical components of the quality of life in patients whose intensity of disability is at a low level was more influenced by mental components like depression. This means that, even with a slight physical disability, mental dimension can be the main determinant of the quality of life in MS patients. Also, psychological disorders like depression may prevent the growth of coping skills and acceptance of the disease (D’Alisa et al., 2006).

In this research the highest and the lowest average scores of social capital belonged to groups and networks and trust and solidarity dimensions respectively. This result is different from the study about cardiovascular diseases, diabetes, and obesity (Holtgrave & Crosby, 2006; Scheffler et al., 2008). It seems that the maximum average score of membership in groups and networks (the structural dimension of social capital) is justifiable because of the dominant features of individuals under study such as gender (females), age group, marital status (single) and level of education (university). “Gender” is an effective variable on social capital and influences even the type of membership in networks. In this regard, women are willing to join the service-charity networks, churches, schools, and cultural and people’s communities while men are interested to join political networks (R. D. Putnam, 1995). “Age” is also an effective factor on the formation of social capital. Some studies have shown that there is a relation between age, sociability, and social participations. From 18 to 29 years old memberships are single while from 30 to 59 years old they are multiple and individuals above 60 years old have less participation in networks (R. D. Putnam, 1995). “Level of education” is another effective variable on the formation of social capital. More participation in networks and social networks are observable among individuals with college education and social relations are common among the educated. In fact, there is a direct relation between official educational years and social capital and this is due to the institutional environments of schools and universities which encourage people to sociability and participating in social networks (R. Putnam, 2012). Therefore, since most participants of this study were women (73 percent) between 30 to 59 years old (60 percent) single (60 percent) and with university education (62 percent), the maximum average score of participation in networks and groups among individuals is justifiable.

Analyzing with multiple regression model in this study showed that the existence of a comorbid disease has a negative effect on the score of combined dimensions of the quality of life as the existence of a comorbid disease caused 0.32 and 0.45 (standardized regression coefficients that in cases where there is more than one effective variable it allows the comparison of their effect size/power) reduction in the score of patients’ quality of life in physical and mental health dimensions that is an expected effect. There is a direct and significant relation between using and not using corticosteroids (including Betaferon, Avonex, Rebif) and the combined dimension of the physical health of the quality of life. As it is observed, using aforementioned medications as opposed to not using them, leads to a 0.37 increase in the score of physical health dimension of quality of life in MS patients. It seems this result is in line with the results of the studies which proved consuming corticosteroids reduces relapse and EDSS (Etemadifar, Janghorbani, & Shaygannejad, 2006; Zimmermann et al., 1999) significantly and consequently improves and enhances the quality of physical life.

In this research, complementary and alternative medicine variable has been effective on the combined dimension of mental health of the quality of life as using the aforementioned medicine leads to a 0.33 increase in the mental health dimension of the quality of life in MS patients. This result is in line with the studies which have shown complementary and alternative medicine is effective on the quality of life (Esmonde & Long, 2008; Nayak, Matheis, Schoenberger, & Shiflett, 2003; Petajan et al., 1996). Complementary medicine is effective on the overall health and quality of life in MS patients by reducing anxiety, depression, increasing general well-being (Petajan et al., 1996) and improving mental health, increasing the adaptability of psychological, nervous, immune, and cognitive systems and modifying immune system (Parshad, 2004).

The significance of the effect of membership in groups and networks variable (structural index of social capital) on the combined dimension of mental health of the quality of life is another finding of this research. As it is observed, every one-unit increase in membership in groups and networks adds 0.38 to the score of quality of life in the mental health dimension. This is true for empowerment and political action dimension but with a more standardized regression coefficient (Sβ = 0.50). This finding is in line with the results of other studies (De Silva, McKenzie, Harpham, & Huttly, 2005; Soltani & Jamali, 2008) which investigated and confirmed the relation between social capital and social networks and their effect on psychological health. This relation is justifiable based on network theory. A network perspective in social capital emphasizes horizontal and vertical relations among people in social groups and societies. Proponents of network theory maintain that social networks function like a fender against internal pressures as they provide emotional support, friendships, and opportunities for significant social actions in the form of social capital and they have an important and influential effect on people’s self-esteem, increase the ability to tackle problems and depressions, and finally lead to the feeling of psychological health (Berkman, 2001).

The collective action and cooperation dimension is also an effective variable on the combined dimension of mental health. Negative standardized regression coefficient shows the negative and reverse relationship between collective action and cooperation dimension with health. This finding is accord with other studies that revealed a non significant and even reverse relationship between social capital and health (Veenstra, 2000; Veenstra et al., 2005). Studies conducted by Ziersch (2005) and Ziersch and Baum (2004) showed that majority of medical scientists are intend to look at positive side of social capital while it’s negative effect (dark side) is ignored. There is the theoretical underpinning for justification of this issue in the literature. Pearce et al., for instance, stated that intervention in societies to increase the level of social capital may lead to its inefficacy and create frustration and over-consuming the resources. Based on such an approach, the individual may be blamed and the effect of social and economic policies at the macro level of physical and psychological health may be ignored (Pearce & Davey Smith, 2003). This stance of social capital in the health area focuses a lot on a micro level and by putting the responsibility on the shoulders of the individual and forcing them to join groups and participate in the society, neglect many of socio structural factors which may facilitate the disease and destroy physical and psychological health and on the other hand the role of the government and other private/public institutions may be neglected.

### 4.1 Study Strengths and Limitations

One of the strong points of this study is that it generally helps social epidemiology and specifically the knowledge in the context of quality of life in MS patients. It seems this is the first study which measures the role of socio contextual factors like social capital on the quality of life of MS patients. Unlike other studies which used only one or two items to measure cognitive-structural social capital, this study used a six dimension tool with 39 questions to measure this multifaceted concept better. It is noteworthy that in this research patients referring to the Iran Multiple Sclerosis Society residing in Tehran were not limited to a special group of patients and, given the services of this center, its members belong to different socioeconomic classes and it seems serious selection bias doesn’t threaten this study.

This research is not without limitations and, like all cross-sectional studies; it is not possible to determine the direction of the relation and causality. Although this research studied the relation of social capital and quality of life, the exact nature of biological or causal mechanism of the relation is not clear and while we assumed higher social capital improves quality of life, better quality of life may increase the individual’s participation for improving social capital in a society. Thus, longitudinal studies or comprehensive cross-sectional ones may clarify the causal direction. Also, since this study relies on self reporting, it may be exposed to reporting bias because, given the subjective nature of measurement, every interviewee may have different understandings of the social capital concept especially the cognitive dimension (norms, values, attitudes) and therefore different answers may be obtained.

## 5. Conclusion

One of the important variables effecting quality of life is the level of social capital. Improving the level of people’s social capital is an equal situation for other effective variables can lead to the remarkable improvement in people’s quality of life. The results of this study showed that the quality of life in psychological health dimension and social capital of MS patients are closely dependent and have a positive effect on each other. In other words, the increase in social capital leads to the improvement of an individual’s quality of life and vice versa. The increase in social capital components of these individuals can on one hand help the society use their abilities and on the other hand it has an important role in their physical and psychological health and consequently the improvement of their quality of life. Therefore, it requires the all-inclusive attention and long-term planning on the side of authorities based on community based research for improving quality of life and fundamental changes for increasing social capital and participation of these patients.
